# Serologic screenings for H7N9 from three sources among high-risk groups in the early stage of H7N9 circulation in Guangdong Province, China

**DOI:** 10.1186/1743-422X-11-184

**Published:** 2014-10-23

**Authors:** Jie Wu, Lirong Zou, Hanzhong Ni, Lei Pei, Xianqiao Zeng, Lijun Liang, Haojie Zhong, Jianfeng He, Yingchao Song, Min Kang, Xin Zhang, Jinyan Lin, Changwen Ke

**Affiliations:** Center for Disease Control and Prevention of Guangdong Province, Guangzhou, 511430 China; International Dialogue and Conflict Management, Vienna, Austria; Guangdong Provincial Institute of Public Health, Guangzhou, 511430 China

**Keywords:** Influenza virus, H7N9 virus, Antibody, Serologic screening

## Abstract

**Background:**

The aim of this study was to assess the prevalence of the novel avian influenza A virus (H7N9) in three high risk groups. The groups were divided into those exposed through infected individuals, those exposed through poultry and those individuals exposed through the external environment, in the early stage of the epidemic in Guangdong Province, which is located in the southern region of China.

**Methods:**

Serologic studies were conducted among samples collected from individuals who had close contact with the first H7N9 infected patient reported in Guangdong Province, those who were most likely exposed to the first group of H7N9 infected poultry, and those who might have been exposed to H7N9 in the environmental settings, namely hemagglutinin inhibition (HI) and microneutralizaiton(MN) assays using three viruses as antigens.

**Results:**

The alignment results of the viral sequences indicated the similarity of the HA gene sequence among viruses from exposure to infected poultry, infected humans and contaminated environments were highly conserved. Seven samples of individuals exposed to contaminated environments were positive in the HI assay and one sample among them was positive in the MN assay using poultry H7N9 virus as the antigen. One sample was positive against human H7N9 virus and 3 samples were positive against environmental H7N9 among those that were in contact with infected patients in HI assay. None of these were positive in MN assay. All HI titers of the 240 samples from those individuals in contact with infected poultry were less than 40 aganist the antigens from three viruses.

**Conclusions:**

The results suggest that when the H7N9 virus was in the early stages of circulation in Guangdong Province, the antigenic sites of the HA proteins of the H7N9 strain isolated from different hosts were highly conserved. The risk of new infection is low in individuals who have contact with the infected patients, poultry or a contaminated environment in the early stages of the circulation of the H7N9 virus.

## Background

In March 2013, a novel reassortant avian influenza A subtype H7N9 virus was first detected from patients with severe respiratory disease in Eastern China [1]. Sequencing analysis revealed that the novel virus was of avian origin with six internal genes from avian influenza A (H9N2) viruses, while the haemagglutinin (HA) and neuraminidase (NA) genes were most likely from Eurasian lineage of avian A subtype H7N3 and H11N9 [2]. Through April 2014, a total of 411 human infections with 147 fatalities were reported in 14 provinces and regions including Taiwan and Hong Kong. During this time, novel H7N9 strains continued to emerge in poultry and in the environment in several provinces in China.

Guangdong, a southern province of China with a sub-tropical climate, is one of the epicenters of the circulation of novel influenza viruses [3]. In April 2013, the H7N9 virus was first detected in poultry in Guangdong, and was then detected in environmental samples in May of that same year. The first human infection case of H7N9 within the province was in July 2013. By July 2013, the novel virus had been detected in all possible vectors of the transmission pathway and had established its circulation in the province.

As a novel reassortant virus, the transmission and infection of H7N9 virus remained inconclusive [4]. Both the epidemiologic and genetic analysis have revealed that close contact with infected poultry or a contaminated environment may be an important risk factor for H7N9 infection in humans [5, 6]. The limited and non-sustainable person-to-person transmission of this novel avian flu virus was reported in Eastern China [7]. As we had identified H7N9 strains from all sources of possible viral infection, namely virus isolated from infected humans, infected poultry and environmental samples, we were able to investigate the ability of H7N9 to transmit from poultry to humans, from a contaminated environment to humans, and from person to person respectively. 561 samples were collected from these high-risk groups at the early stage of viral infection. Serological studies of these samples provide useful information on the circulation of H7N9. Here we report the results of the serological screenings conducted on samples collected from individuals in contact with infected patients, individuals exposed to infected poultry, and individuals exposed to environments contaminated with H7N9 virus, in the early rise of the epidemic in Guangdong Province, Southern China.

## Results

### The detection and sequence of H7N9 virus

Samples taken from poultry cages in live poultry markets were detected to be positive for H7N9 in May 2013. This was the first time that the H7N9 virus was detected in an external environmental sample in Guangdong Province. The first H7N9 infected patient in Guangdong Province was confirmed with real-time PCR, of the H7 and N9 subtype, in July 2013. The confirmed patient, a woman of 56 years, was responsible for sales and butchering of poultry in a live poultry market for ten years. She was reported to have killed and sold an average of 10 chickens and 5 ducks per day before onset of symptoms. These two H7N9 viruses (from the patient and the environmental sample) were identified as A/Huizhou/1/2013(H7N9) and A/environment/Guangdong/25/2013 (H7N9), respectively. The whole genomes of these two viruses were sequenced and analyzed.

### Alignment of HA sequence among H7N9 viruses originated from different sources

**Figure 1:**
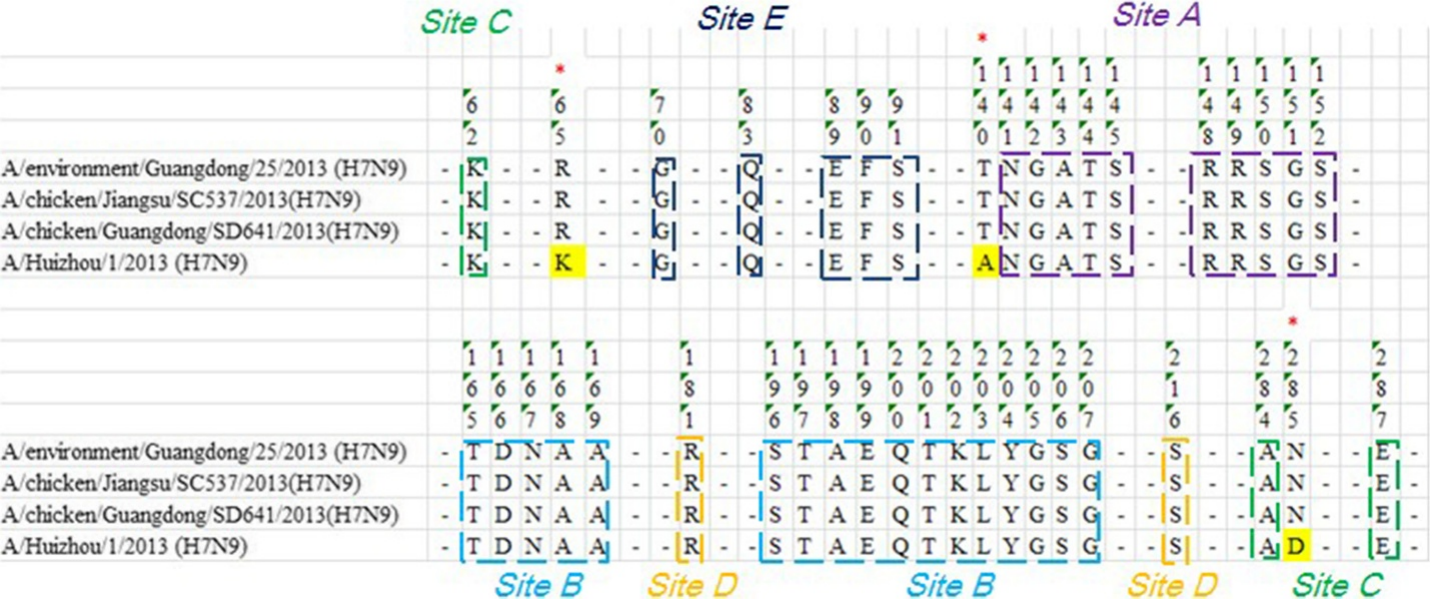
**The alignment of HA protein of 4 viruses from different origins.** The gene sequence of HA of A/Huizhou/1/2013(H7N9) and A/environment/Guangdong/25/2013 (H7N9) was sequenced by GDCDC. The gene sequence of HA of A/chicken/Jiangsu/SC537/2013 (H7N9) [GenBank:CY146940.1] and A/chicken/SD641/Guangdong/2013 [GenBank:CY146908.1] were retrieved from GenBank. The similarity of the nucleotide sequence was 99.5-99.8% to the HA gene and there are 3 different amino acids sites of R65K, T140A and N285D of the HA protein among the 4 viruses.

The HA gene of the viruses originating from the patient, identified as A/Huizhou/1/2013 (H7N9) [GISAID: EPI439507] and the environmental sample, identified as A/environment/Guangdong/25/2013 (H7N9) [GenBank: KF667746.1] were sequenced by GDCDC. The HA gene of viruses isolated from poultry, identified as A/chicken/Jiangsu/SC537/2013 (H7N9) [GenBank:CY146940.1] and A/chicken/SD641/Guangdong/2013 [GenBank:CY146908.1] were retrieved from GenBank [8]. The similarity of nucleotide sequence is 99.5-99.8% with the HA gene and there are 3 different amino acids sites of R65K, T140A and N285D of the HA protein among the 4 viruses (Figure 1).

### Epidemiological findings

A total of 125 serum samples were collected from individuals in close contact with the index patient who was the first identified patient infected by H7N9 in Guangdong Province. These individuals ranged from family members, neighbors, healthcare personnel, workers from the live poultry market and slaughterhouse, to those who had social contact with the patient. The median age of the 125 individuals was 38 years (1–74 years), with 36.8% male (Table 1).

**Table 1:** **Gender and age of 125 individuals who were in contact with the first identified H7N9 infected patient**

	Healthcare personals	Family members	Poultry market and slaughterhouse	Social contact	Neighbors
**Male**	2	6	8	6	24
**Female**	4	7	9	14	45
**total**	6	13	17	20	69
**Median age (years [interquartile range])**	40 [27–43]	35.5 [8–74]	36.5 [15–61]	38 [3–64]	40 [1–70]

A total of 240 serum samples were collected from those who had possible exposure to the first H7N9 infected poultry in Guangdong Province, including staff from live-poultry markets, farms, poultry yards, slaughterhouses, transports, individuals from the Animal Health Inspection Agency and the family members of the poultry market staff. The median age of these 240 individuals was 41 years (1–72 years), with 67.5% male (Table 2).

**Table 2:** **Gender and age of 240 individuals with possible exposure to the first identified H7N9 infected poultry**

	Poultry meat vender and their family members	Other venders in the same market and their family members	Other workers in slaughterhouse	Other workers in live poultry market	Animal Health Inspection Agents	Egg sales
**Male**	71	24	7	23	34	3
**Female**	33	16	7	12	3	7
**Total**	104	40	14	35	37	10
**Median age (years [interquartile range])**	41 (2–65)	41.5 (1–72)	42 (21–52)	41 (22–60)	36 (27–56)	43 (16–50)

A total of 196 single serum samples were collected from individuals who might have exposure to the environment that was contaminated with H7N9 early in the epidemic, ranging from live poultry market managers, lodging clerks and cleaning staff, to poultry meat vendors and butchers. The median age of this group was 42 years (11–64 years) with 72.4% male (Table 3).

**Table 3:** **Gender and age of 196 individuals with possible exposure to a contaminated environment**

	Transportation workers	Market staffs	Poultry sales and their households
**Male**	17	10	115
**Female**	0	4	50
**Total**	17	14	165
**Median age (years [interquartile range])**	40 [22–59]	47 (29–63)	42 [11–64]

At the time when blood samples were taken, none of the 561 individuals showed no influenza symptoms. All participants were taken throat swabs which were tested negative for nucleic acid of H7N9 virus with RT-PCR.

### Serological results

The 561 samples were screened for the antibodies specific to influenza A/Huizhou/1/2013 (H7N9), A/chicken/Jiangsu/SC537/2013 (H7N9) and A/environment/Guangdong/25/2013 (H7N9). With a conservative cut-off value, the antibody titer higher than 40 was considered as a positive result. The serology results of 561 serum along with patient and positive control are show in Table 4. Four serum samples, from the patient’s husband, employees and daughter respectively, were positive against A/Huizhou/1/2013 (H7N9) and A/environment/Guangdong/25/2013 (H7N9) using the HI assay. Seven serum samples from vendors in a live poultry market where the H7N9 virus was first found on chicken cages and the ground were positive for avian influenza virus (H7 Sub-type). Only one sample of an individual exposed to a contaminated environment was positive with the confirmed MN assay among the 11 positive samples using HI (Table 4).

**Table 4:** **HI results of the serological screening of 561 specimen with three different viruses**

	A/Huizhou/1/2013	A/chicken/Jiangsu/SC537/2013 (H7N9)	A/environment/Guangdong/25/2013
**Individuals in contact with infected person**	1	0	3
**Individuals exposed to infected poultry**	0	0	0
**Individuals exposure to contaminated environment**	0	7	0
**HI titer of serum from infected patient**	1:80	1:160	1:80
**HI titer of positive control**	1:80	1:40	1:160

## Discussion

Antigenic drift or shift of Influenza viruses takes place when the virus is exposed to various hosts or vectors in nature. Antigenic drift is related to climate, location and population [9, 10]. In order to elucidate the antigenic drift, transmission and infection in the population of the novel H7N9 virus at the onset of the epidemic and circulation in southern China, three different origins of the H7N9 virus isolated from key sources of transmission were selected as antigens to detect the antibody to H7N9 in sera of the high risk groups who had close contact with the first human case of H7N9, those, who had close contact with infected poultry, or exposure to H7N9 contaminated environmental settings.

HA is the major glycoprotein envelope of the influenza A virus and the target of almost all neutralizing antibodies [11]. There are five antigenic epitopes (epitope A, B, C, D and E) in the HA1 subunit and in most subtypes of influenza A [12]. Most neutralizing antibodies bind to the exposed loops of the receptor binding sites of HA blocking the binding of the virus to cell receptors [13–15]. Because these loops are highly variable, the amino acid sequence of related antigenic sites of the five H7N9 viruses identified in the human, poultry and environmental samples were analyzed. There are 3 different amino acids sites of R65K, T140A and N285D of the HA protein among viruses isolated from humans, poultry and the environment in the HA protein. This indicates that viral mutation has already occurred when changing host during the early circulation of the virus in Guangdong Province. Although the three mutant sites do not locate in epitopes, K65R and N285D are both close to epitope C and site T140A is close to eptiope A, which would be helpful to the advanced protein structure for the binding antibody. Thus cross-reactivity of viruses originating from the three different sources was not found in HI. However, the similarity of nucleotide sequences of the HA gene of the three H7N9 strains from the different origins was 99.5-99.8% indicated that the antigenic drift of the H7N9 virus was low at the early stage of the infection in Guangdong Province.

To study the possible transmission routes of H7N9, we screened the serum samples collected from the high-risk groups (individuals in close contact with the index patient and infected poultry, or exposure to a contaminated environment) against three H7N9 viruses that originated from poultry, human and the environment. We intended to dissert the possible route of transmission by serologically screening these samples. However, our results indicated that the positive rates of antibodies against H7N9 viruses of three different origins were very low within the high-risk groups. This might suggest that the infection rate of H7N9 virus was very low within those that were in close contact with the infected H7N9 patient, infected poultry or contaminated environment when H7N9 was first circulating in Guangdong Province. The transmission of H7N9 from person to person, from poultry to person, and from environment to human might be low in the early stage of viral circulation in Guangdong Province which is located in a subtropical climate, which is a humid environment that will favor the survival of the virus.

There are several reports that are consistent with our findings in this study. Hsieh SM et al. reported that the HI activity of serum from 14 close contacts to the first confirmed case of H7N9 infection in Taiwan were all <1:10 and none of the close contacts had flu-like or respiratory symptoms during the 28 days of follow-up. This provided evidence that the potential for person-to-person transmission of H7N9 was low [16]. Li et al. reported a serologic screening study of 1251 close contacts of 82 H7N9-confirmed patients in China. They found 19 cases of respiratory symptoms from these close contacts but all cases tested negative for the H7N9 virus [5]. Qiu et al. reported that all family members of three patients infected by H7N9 showed negative reactions on H7N9 viral tests, although they all had close contact with the patients during the viral shedding period [17]. All of these previous reports indicated that the novel avian-origin H7N9 virus was not easily transmitted by close contact of H7N9 infected patient.

Interestingly, the highly pathogenic avian influenza A (H5N1) virus that circulated among poultry is also known to not be easily transmitted by close contact with infected patients [18, 19]. Although H7N9 virus binds to both receptors of α-2-6 sialosides and α-2-3 sialosides, it speculated that the cell binding pattern to a cell receptor of H7N9 is similar to of the H5N1 [20].

Serologic study on 396 poultry workers identified >6% sero-positive for H7N9 (on the basis of HI titer of ≥80), suggesting that infected poultry might be a principal source of human infections in Zhejiang Province [21]. Yet our study showed low sero-positive cases from the same group of workers, resulting in a large difference in the sero-positive rate of H7N9 in the Provinces of Zhejiang and Guangdong. This might be due to the difference in the timing of sample collection. The majority of the confirmed cases of H7N9 were reported in Zhejiang Province in 2013 and samples from poultry workers were collected after 45 cases of H7N9 were confirmed. At the time of sample collection, H7N9 or a closely related virus may have already been in circulation in live poultry markets in the region for a period of time in Zhejiang Province. The samples in this study were collected in the early phase of viral circulation in Guangdong Province. Thus, the timing of the viral circulation was different in the two studies, one from the established stage and one from the early stage, which may have lead to the difference in sero-positive rate among the high-risk groups of individuals.

It is worth mentioning that the serological screenings for H7N9 among high-risk groups in Guangdong Province should be continued in order to monitor sero-positivity and to support the hypothesis drawn from the experimental results.

## Conclusions

Taken together, our findings suggest that in early stage of epidemic of the novel H7N9 virus in Guangdong Province, the sequence of the HA protein of H7N9 strains from different hosts were highly conserved. The infection rate of the H7N9 virus is very low among high-risk groups. Proactive screening of individuals from the high-risk groups of viral infection may provide clues of the evolution of the virus in endemic regions.

## Methods

### Detection, isolation and sequence of H7N9 virus

Specimens from swabs from humans, poultry and environmental samples were detected with real-time PCR (The Avian influenza virus H7N9 Real Time RT-PCR kit manufactured by Shanghai ZJ Bio-tech Co, LTD). The viruses were isolated with 9-11-day-old specific pathogen free (SPF) embryonated chicken eggs and allantoic fluids were collected and sequenced. Eight fragments from H7N9 positive samples were amplified using PathAmpTM Flu A Reagents (Applied Biosystems, Foster City, CA) and sequenced with the Ion torrent Personal Genome Machine (Applied Biosystems, Foster City, CA). Sequence analysis was performed using MEGA version and the alignments were conducted with the software MEGA5.0 with MUSCLE Algorithm.

### The virus and viral inactivation

Three avian influenza H7N9 viruses were selected to screen the samples collected from the early stages of viral infection. A/environment/Guangdong/25/2013 (H7N9) was isolated from the environmental samples detected at the earliest stage of the epidemic in Guangdong Province. A/Huizhou/1/2013 (H7N9) was isolated from the first infected patient of Guangdong Province. A/chicken/Jiangsu/SC537/2013 (H7N9) was isolated from infected poultry purchased from Harbin Weike Biotechnology Development Ltd. All three viruses were propagated in SPF grade embryonated chicken eggs in a Bio-Safety Level (BSL) 3 facility and were used as the probing antigen in HI and MN assays. The serum containing H7N9 specific antibody used as positive control was obtained from the Chinese Centers for Disease Control (CDC). The harvested virus was inactivated by beta-propiolactone treatment (37°C for 2 hours). Receptor destroying enzyme II (RDE II) produced from *Vibrio cholera* serovar 558 Ogawa was purchased from DENKA SEIKEN Ltd. The 96-well plates were purchased from Greiner Bio-one Suns Ltd.

### The definition of high-risk groups

The high-risk groups of individuals were defined as the following:

Those who had close contact with the infective patients (such as household contacts and social contacts that had contact within one meter of the patient), and those who had direct contact with the patient (such as clinical staffs).

Those with possible exposure to poultry infected with H7N9, including those who sold and butchered poultry, and those who worked in the live poultry market.

Those who may have been exposed to a contaminated environment, such as those who were exposed due to lack of effective personal protective equipment,. These include workers in poultry farms, slaughterhouses, live-poultry markets, and those who are involved in transportation of poultry.

### Serum sample collection

Once the first H7N9 infections in human, poultry and the environment were confirmed in Guangdong Province, an investigation team composing of the staffs from both local CDC and provincial CDC was formed to immediately examine any possible source of the H7N9 virus. The serum samples of high-risk groups were collected to investigate the possible routes of transmission. All blood specimens were collected by venipuncture and placed on ice packs and transported to the local CDC laboratory for further processing. Samples from individuals who had close contact with the first H7N9 infected patient were collected 20 days later (after initial patient symptom onset) and samples from those who were exposed to infected poultry and environmental sources were collected 2–3 weeks after the initial infection. Serum was separated, and split into three aliquots and temporarily frozen at -20°C at the local CDC laboratory before shipping to the provincial CDC laboratory on dry ice within 4 days. The samples were then stored at -70°C before further assays were performed.

For the collection and use of all above-mentioned specimens, written informed consent from all participants (their parents or legal guardian in the case of a minor) involved in the research were obtained. This study was approved by the ethics committee of the Guangdong Provincial Center for Disease Control and Prevention, and was in compliance with the Helsinki Declaration.

### Serological screening on the selected samples

An HI assay using horse red cells was employed for H7N9 specific antibody screening on all collected samples in a BSL 2 facility. Serum with an HI titer higher than 40 was defined as seropositive [22] and then subject to an MN assay in an enhanced BSL 3 facility.

Horse RBC solution of 1% (vol/vol) was prepared by washing with PBS containing 0.5% BSA. The serum was treated with RDE (destroying non-specific receptor) at 1:4 (vol/vol) at 37°C for 19 hours, and then heated inactive at 56°C for 30 minutes. 20 μl concentrated horse RBCs was added to 200 μl RDE-treated serum for 1 hr at RT, which could remove the non-specific HI effects. After centrifugation at 2000 rpm for 3 min, the prepared serum (25 μl of each) was diluted in two-fold serial dilutions with 25 μl PBS in 96-well V-bottom microtiter plates. Subsequently, 25 μl of beta-propiolactone inactivated the H7N9 virus containing 4 HAU was added to the wells and incubated at room temperature for 20 minutes. Then, 50 μl of 1% horse RBCs was added and the plate was incubated further for 1 hour at room temperature before the agglutination titers were recorded. The HI assay results were expressed as the reciprocal of the last dilution of the sample that completely inhibited haemagglutination. All assays included samples for the positive serum control.

The positive serum samples were subjected to MN. 10 μl of heat-inactivated sera including positive serum, negative serum and serum samples were added to 96 well cell culture plate (Nunc Corp) and performed 2-fold serial dilutions. 100TCID_50_/50 μl virus was added to wells and we incubated the virus-serum mixture for 1 hr at 37°C, 5% CO_2_. Back-titration, started with the virus test dilution (100 TCID50) and an additional serial 2-fold dilutions with diluents was set-up. 100 μl MDCK cells (1.5 × 10^4^ cells /well) was then added to each well and the plate was incubated overnight at 37°C, 5% CO_2_ (18–20 hrs). The plate was fixed with 100 μl/well of cold fixative at RT for 10 min. The virus was detected with anti-NP monoclonal antibody (KPL Company) and HRP-goat anti-mouse IgG (SANTA CRUZE) as secondary antibody using ELISA. The value below X(X = (Average OD of Positive cell control wells-Average OD of negative cell control wells)/2 + (Average OD of negative cell control wells)) was positive for neutralization activity.

### Ethical approval

For the use of all above-mentioned specimens of serum, written informed consent from all participants (their parents or legal guardian in the case of a minor) involved in the research were obtained. And this study was approved by the ethics committee of the Guangdong Provincial Center for Disease Control and Prevention, and was in compliance with the Helsinki Declaration.
